# Multiple imputation strategies for zero-inflated cost data in economic evaluations: which method works best?

**DOI:** 10.1007/s10198-015-0734-5

**Published:** 2015-10-23

**Authors:** Janet MacNeil Vroomen, Iris Eekhout, Marcel G. Dijkgraaf, Hein van Hout, Sophia E. de Rooij, Martijn W. Heymans, Judith E. Bosmans

**Affiliations:** Academic Medical Center, University of Amsterdam, Amsterdam, The Netherlands

**Keywords:** Cost data, Economic evaluation, Missing data, Multiple imputation

## Abstract

Cost and effect data often have missing data because economic evaluations are frequently added onto clinical studies where cost data are rarely the primary outcome. The objective of this article was to investigate which multiple imputation strategy is most appropriate to use for missing cost-effectiveness data in a randomized controlled trial. Three incomplete data sets were generated from a complete reference data set with 17, 35 and 50 % missing data in effects and costs. The strategies evaluated included complete case analysis (CCA), multiple imputation with predictive mean matching (MI-PMM), MI-PMM on log-transformed costs (log MI-PMM), and a two-step MI. Mean cost and effect estimates, standard errors and incremental net benefits were compared with the results of the analyses on the complete reference data set. The CCA, MI-PMM, and the two-step MI strategy diverged from the results for the reference data set when the amount of missing data increased. In contrast, the estimates of the Log MI-PMM strategy remained stable irrespective of the amount of missing data. MI provided better estimates than CCA in all scenarios. With low amounts of missing data the MI strategies appeared equivalent but we recommend using the log MI-PMM with missing data greater than 35 %.

## Introduction

Researchers should aim for collecting high quality and complete data, as missing data may lead to loss of information in epidemiological and clinical research [1]. However, missing data are unavoidable when performing trials where data is collected through self-report by the participants. Cost data are prone to missing data because economic evaluations are often “piggy-backed” onto clinical studies where cost data are rarely the primary outcome. Moreover, one missing cost measurement results in a missing total cost estimate, because costs are summed over all measurements.

Three types of missing data are commonly distinguished; missing completely at random (MCAR), missing at random (MAR) and missing not at random (MNAR). MCAR refers to data that is missing by chance and is unrelated to the study participants. An example of MCAR is a questionnaire that is accidentally lost in the mail. Data MCAR do not bias the results of the study, but do decrease the power of the study. Missing at random (MAR) occurs when there is data that is missing from the data set, but there are variables in the data set that can explain why the data is missing. As we know the reason for the missing data, we can create models to fill in this missing data. Missing not at random (MNAR) is where there is data that is missing and there are no variables to explain why the data is missing. An example of this could be that participants who work full-time do not return questionnaires because they are too busy. However, we do not have information available on the number of hours worked by participants. If this characteristic is also related to the outcome of interest, the results of the study will be biased. Imputation of data is difficult because no information is available that predicts missingness of data.

Complete case analysis (CCA) is the default strategy to deal with missing data although it is known for biased estimates, wide standard errors and decreased power. Oostenbrink et al. [2] and Briggs et al. [3] showed that multiple imputation techniques performed better than CCA and simple imputation techniques [conditional mean imputation, single imputation with predictive mean matching (PMM), hot decking and expectation maximization] [2, 3].

Recently, multiple imputation has been recommended as the most appropriate way for handling missing data [1, 4–7]. Multiple imputation can be a powerful tool for estimating missing data [5], but there are some important points to consider when specifying the multiple imputation model. First, the imputation model should include all variables that explain missing values. Second, it should include all variables included in the analysis model, and third the imputation model must account for the distribution of the data. This assumption may not be met when imputing cost data in trials because of the distributional issues posed by cost data, including constrained positive values, a large amount of zero values, and right-handed tail skewness.

Multiple imputation with predictive mean matching (PMM) can be a helpful tool for dealing with the skewed distribution of cost data, because PMM preserves the distribution of the data and, therefore, is robust against violations of the normality assumption [5]. Another commonly recommended approach for dealing with skewed data is to take the log of the skewed variables before imputation and then back transform the variables to their original scale before the target analysis [4, 5, 8]. Lee and Carlin [8] compared multiple imputation with transformation and PMM to deal with non-normality in continuous variables. They recommended transformation of skewed variables to a symmetric distribution to avoid the introduction of biases of study results. Another alternative is to impute missing data in two separate steps. In the first step, the probability of having costs is imputed which takes care of the zero inflation, and in the second step, an actual cost value is imputed for individuals that are predicted to have costs. In the second step, the skewness of the cost data is taken into account by using the PMM algorithm to impute the cost values for the people that are predicted to have costs using only the observed cost data [9].

It is unclear which method to deal with imputation of skewed data is the most appropriate in economic evaluations. Therefore, the objective of this article was to investigate which imputation strategy is most appropriate to impute missing cost and effect data in an economic evaluation alongside a pragmatic randomized controlled study. The strategies compared include complete case analysis (CCA), MI with predictive mean matching (MI-PMM), MI with predictive mean matching on log-transformed costs (log MI-PMM), and two-step multiple imputation with predictive mean matching (two-step-MI).

## Methods

### Reference data set

The reference data set was obtained from two open-labelled randomized controlled trials evaluating the cost-utility of medical co-prescription of heroin compared with methadone maintenance treatment alone among 430 chronic, treatment resistant heroin addicts with a follow-up period of 1 year. Psychosocial treatment was offered throughout the trials. Full details on this study are presented elsewhere [10]. Outcomes included QALYs based on the EuroQol (EQ-5D) and costs from a societal perspective [11]. The EQ-5D includes five dimensions: mobility, self-care, usual activities, pain/discomfort and anxiety/depression [11]. The respondent answers each of the EQ-5D’s five dimensions with one of three possible responses: ‘no problems’, ‘some problems’ or ‘severe problems’. Each participant completed the EQ-5D at baseline and at months 6, 10, and 12 during treatment. The health states from the EQ-5D were subsequently converted to utilities using the York tariff [12]. We calculated QALYs by multiplying the utility of each health state by the time in between two measurements and summing the results over the 12-month treatment period. Cost estimates were measured through clinical report forms and the European version of the addiction severity index (EuropASI) [13] to collect data on the use of healthcare resources, travel related to the programme and illegal activities. The EuropASI was completed at the same intervals at the EQ-5D. The valuation of the cost categories was according to Dutch guidelines [14]. Occasional missing values were imputed using last observation carried forward, resulting in a complete data set for all 430 participants which was considered the complete reference data set.

### Missing data

Author 2 generated missing data in the complete data set using R statistical software [15, 16]. With this program [16], multivariate incomplete data can be generated according to the MAR mechanism, which means that the creation of missing data is independent of the imputation models that were evaluated. We used a linear combination of the observed data to get the probability of having missing data for each person in the data set.

Three incomplete data sets were created with 17, 35 and 50 % missing data to investigate the effect of the rate of incomplete data on the performance of the imputation methods. We chose these percentages to reflect low, medium and high amounts of missing data that might influence the results of the analysis [17]. It has been shown that missing data under 10 % will not affect the results of the analysis considerably [17]. Even with 50 % missing data, multiple imputation can result in valid inferences on the data [5].

Missing data points were created in the QALY variable and several cost variables. The probability of missing data was related to other variables in the data to satisfy a missing at random (MAR) assumption for the missing data. Centre, location, age, administering of a second interview, and abstinence were predictors of missingness in the utility and cost outcome variables for the data set with 17 % missing data. In the data set with 35 % missing data, predictor variables were treatment group, centre, sex, age, and occurrence of a second interview. In the data set with 50 % missing data, the predictor variables were treatment group centre, age and occurrence of a second interview. Table 1 presents all key cost variables with missing data for the different missing data scenarios.

### Missing data strategies

#### CCA

In CCA, analysis was restricted to participants with complete cost and effect data. This resulted in smaller sample sizes than in the reference data set (see Table 1).

#### Multiple imputation procedure

Multiple imputation was done using fully conditional specification. Fully conditional specification or chained equations is a flexible multivariate model that does not rely on the assumption of multivariate normality [5]. Regression models are specified for each variable with missing values, conditional on all of the other variables in the imputation model. Imputations are generated by drawing from iterated conditional models [5].

The imputed values were estimated using the predictive mean matching (PMM) algorithm. PMM is an algorithm that matches the missing value to the observed value with the closest predicted estimate [4]. The predicted mean is estimated in a regression equation where a random residual term is added to the estimate in order to account for missing data uncertainty. In PMM, instead of using the predicted estimate, the imputed value is randomly selected from observed values that are closest to the predicted estimate. For example, an older single man misses a measurement for blood pressure and the value for this man is estimated to be 102.34 mmHg by regressing blood pressure on age and sex. Five other older single men have observed blood pressures of 103; 103; 102; 101, and 104 mmHg, respectively. The missing value is then imputed with a random draw from these five blood pressures. PMM has several advantages when imputing cost data. It is more robust against non-normal data as it uses the observed distribution of the data. Furthermore, it imputes only plausible values because it randomly draws from observed values. The process of estimating imputed values is repeated in sequential cycles, each time using the updated data with the imputed estimates from the previous cycle. These cycles are called iterations. One of these iterations (e.g. the 100th) was selected and used as an imputed data set until ‘m’ data sets were selected in total. We used 200 imputations to minimize internal variation so that the imputation variation would not affect the performance of each imputation method [1, 18–20]. We performed MI using the chained command in Stata 12, which uses fully conditional specification to perform the multiple imputations [21].

We performed the multiple imputations stratified by treatment group to maintain the possible group effect in the data. For all multiple imputation strategies we checked the convergence plots to see if iterations were free from trend, and imputations were successful. To solve any occurring convergence problems, we merged highly correlated variables together. For this reason, travel costs were merged together with total programme costs (correlation coefficient >0.9). In-patient hospital consultations and in-patient length of hospital stay were also highly correlated and were therefore merged together as well.

Three multiple imputation strategies were compared and are described below.

1. MI-PMM: in the first multiple imputation strategy we performed multiple imputation with predictive mean matching on the raw data.
2. Log MI-PMM: in the second multiple imputation strategy, we applied the predictive mean matching algorithm to the log transformed cost data. This was done by first adding a constant to the raw cost data in order to circumvent problems when transforming zero values, and next the log was taken. After imputation, the complete data were transformed back to their original scale prior to any analyses being performed.
3. Two-step MI: the third multiple imputation strategy was a conditional two-step approach. We recoded cost variables to dummy variables where subjects were coded as 1 if they had costs and a 0 for no costs. Missing values were left to be multiply imputed with either a 0 or 1 using a logit function. Next, multiple imputation with the PMM algorithm was performed for missing cases with a value 1 on the dummy variables. Only cases with cost estimates higher than zero were used for this imputation step. For variables that did not have a sufficient amount of zeroes to perform the conditional imputation, we chose to apply only the second step on the raw cost variable.

### Statistical analysis

We used a generalized linear regression model with a gamma distribution and an identity link to estimate mean differences in total costs. The gamma distribution was chosen to take into account the right skewness of the cost data. The generalized linear model for quality adjusted life years (QALYs) was adjusted for baseline utility estimates. Mean differences and standard errors were pooled using Rubin’s rules [20].

We estimated the correlation between the incremental total costs and the incremental QALYs in the reference data set and the imputed data sets. In the multiple imputation strategies, the covariance between total costs and QALYs was calculated based on the Fisher z transformation and was then pooled using Rubin’s rules [5, 22].

Incremental cost-effectiveness ratios (ICERs) were calculated using the pooled cost and effect estimates. The ICER is calculated as $\frac{{\hat{\Delta }_{c} }}{{\hat{\Delta }_{e} }}$, where $\hat{\Delta }_{c}$ is the difference in total costs between the two intervention groups and $\hat{\Delta }_{e}$ is the difference in QALYs between the two intervention groups.

Incremental net benefit (INB) estimates were calculated using the following formula: ${\hat{b}}\left( \lambda \right) = \hat{\Delta }_{e} \lambda - \hat{\Delta }_{c}$ [23, 24], where $\hat{\Delta }_{e}$ is the difference in QALYs between the two intervention groups, *λ* is the willingness to pay, and $\hat{\Delta }_{c}$ is the difference in costs. The variance of INB was calculated using: $V\left[ {\hat{b}\left( \lambda \right)} \right] = \hat{V}(\hat{\Delta }_{e} )\lambda^{2} + \hat{V}\left( {\hat{\Delta }_{c} } \right) - 2\hat{C}\left( {\hat{\Delta }_{e} ,\hat{\Delta }_{c} } \right)\lambda$, where $\hat{C}$ is the covariance between the differences in total costs and QALYs [23, 24]. We set the willingness-to-pay at €30,000 because this is roughly equivalent to the cut-off value mentioned in the Standard National Institute of Clinical Excellence guidelines (₤20,000–₤30,000 per QALY) for economic evaluations [25].

Cost-effectiveness acceptability curves (CEAC) were estimated to quantify the uncertainty due to sampling and measurement errors and because lambda is generally unknown. The CEAC is a plot of the probability that co-prescribed heroin compared to methadone maintenance only is cost-effective (*y*-axis) as a function of the money society might be willing to pay for one additional QALY (*x*-axis). The pooled coefficients and variance parameters from the regression models were used for the CEACs.

### Comparison of strategies

The estimates from the reference data set were considered the “true values” and we compared the estimates from the different multiple imputation strategies with these true values. The primary outcomes of interest were the value of INB at a willingness to pay of €30,000 per QALY, the standard error of INB and the probability that co-prescribed heroin compared to methadone maintenance at a willingness to pay of €30,000 per QALY. We evaluated the percentage of bias from the reference analysis (RA) in the different imputation strategies for cost and effect differences, standard error estimates, *p* values and *t* values. The strategies that gave the closest estimates to the reference data set were considered the best.

### Sensitivity analysis

Research suggests that it is better to impute at the item and not the total level [26, 27]. Therefore, we imputed the total cost variable directly as a sensitivity analysis for all missing data strategies.

## Results

### Costs

Table 2 contains baseline characteristics and the variables used to calculate the utilities. Total costs consisted of programme costs, law enforcement costs, costs of damage to victims, health related travel costs and other health care costs. Table 1 presents the frequency distributions of each cost category in the reference data set and the other multiple imputation strategies. Table 3 presents the cost estimates for the reference case, the CCA, and the different imputation strategies for 17, 35 and 50 % missing data. The difference in costs of −€12,792 in the RA fell within the 95 % confidence intervals of all multiple imputation strategies for all rates of missing data. The CCA deviated the most from the RA compared to all other strategies, specifically with regard to the cost differences and the associated standard errors in all scenarios. For 17 % missing data, the CCA showed a statistically significant difference in costs just as in the reference analysis. However, for 35 or 50 % missing data the cost difference was no longer statistically significant. The multiple imputation strategies gave similar results to each other in the 17 and 35 % missing data sets showing smaller differences in costs and larger standard errors when the amount of missing data increased compared to the reference analysis. The log transformed-PMM deviated the least from the RA in the 50 % missing data set for the cost difference, standard error and *p* values. The two-step MI deviated the most from the RA with regard to cost differences and the standard errors in the data set with 50 % missing data.

**Table 1:** Baseline characteristics of the reference data set

Explanatory variables	Methadone alone (*n* = 237)	Co-prescribed heroin (*n* = 193)
% Male (*n*)	55.1 (190)	44.9 (155)
Age (SD)	38.9 (5.7)	39.7 (5.8)
% Injected (*n*)	56.3 (98)	43.7 (76)
% Completed (*n*)	60.2 (204)	39.8 (135)
% Abstinent (*n*)	59.3 (80)	40.7 (55)
% Second interview performed (*n*)^a^	55.7 (59)	44.3 (47)
Baseline utility (SD)	0.731 (0.273)	0.739 (0.272)

^a^Those included early in the trials also completed the questionnaire in the second month. *SD* standard deviation. Figures are frequencies (column percent)

**Table 2:** Descriptive statistics of the cost variables (euros) for the reference data set and the data sets with missing values

Description	QALY [12] on a 0–1 scale	Travel costs	Total programme costs	Out-of-hospital consult costs	In-patient hospital consult costs	In-patient hospital stay costs	Police investigations of criminal offenders costs	Convicting criminal offenders costs	Sanctioning criminal offenders costs	Damage to victims costs
*Reference data set*										
Mean cost (euros)	0.76	350.04	8692.99	45.97	316.12	778.96	6004.28	3172.95	1854.53	23,602.36
Standard deviation	0.22	292.79	10,315.04	124.06	1206.92	3680.04	14,497.13	12,627.55	5935.78	59,945.50
Percentage of zeroes	N/A	3.3	0.2	67.4	56.7	91.6	58.4	88.8	77.2	71.2
Skewness	−1.28	0.90	0.93	4.81	10.39	7.32	7.10	6.14	4.20	3.17
*18* *% Missing*										
Mean cost (euros)	0.75	350.04	8266.09	43.89	323.21	727.68	6304.52	3260.05	1751.09	24,391.48
Standard deviation	0.23	292.79	10,022.88	109.55	1267.93	3446.62	15,219.27	12,995.47	5797.58	61,348.19
Percentage of zeroes	N/A	3.3	0.3	67.2	58.1	91.8	58.9	88.9	77.8	71.0
Skewness	−1.30	0.90	0.99	3.95	10.01	7.36	6.81	6.06	4.45	3.14
*35* *% Missing*										
Mean cost (euros)	0.75	350.04	8218.24	44.99	336.41	794.57	6462.09	3027.35	1731.97	24,611.35
Standard deviation	0.23	292.79	10,075.05	112.93	1318.40	3711.62	15,665.51	12,569.37	5783.11	62,221.27
Percentage of zeroes	N/A	3.3	0.3	66.0	57.4	91.6	58.8	89.6	78.1	70.7
Skewness	−1.23	0.90	1.02	4.00	9.69	7.09	6.75	6.40	4.56	3.17
*50* *% Missing*										
Mean cost (euros)	0.74	350.04	7907.28	49.09	338.09	853.85	6523.91	2586.35	1749.48	23,280.58
Standard deviation	0.23	292.79	9995.16	118.29	1357.34	3972.24	15,794.79	11,541.70	5873.06	60,485.85
Percentage of zeroes	N/A	3.3	0.3	63.7	57.7	91.0	57.7	90.5	78.2	72.5
Skewness	−1.20	0.90	1.11	3.82	9.60	7.00	6.99	7.31	4.58	3.27

*N/A* not applicable

**Table 3:** Overview of cost estimates for the missing data methods

	RA		CCA (% bias)		MI-PMM (% bias)		Log MI-PMM (% bias)		Two-step MI (% bias)	
	M	M + H	M	M + H	M	M + H	M	M + H	M	M + H
*17* *% Missing data*										
*n*	237	193	201	154	237	193	237	193	237	193
Mean cost (euros)	50,560	37,767	53,148	38,933	51,369	37,935	51,966	38,137	52,685	38,482
SE mean (euros)	5359	3063	6056	3744	5650	3268	5652	3309	5642	3394
Treatment cost difference (euros)	−12,792		−14,215 (11)		−13,434 (5)		−13,829 (8)		−13,203 (3)	
SE cost difference	6086		7077 (14)		6440 (5)		6459 (5)		6506 (6)	
*z* for CCA and *t* for MI	−2.100		−2.010		−2.090		−2.140		−2.030	
*p* value	0.036		0.045		0.037		0.032		0.042	
95 % CI lower limit	−24,720		−28,085		−26,057		−26,489		−25,954	
95 % CI upper limit	−865		−345		−810		−1169		−452	
*35* *% Missing data*										
*n*	237	193	163	122	237	193	237	193	237	193
Mean cost (euros)	50,560	37,767	52,255	43,176	50,810	39,851	51,434	40,408	51,195	40,426
SE mean (euros)	5359	3063	6953	4560	5989	3448	5975	3601	6052	3551
Treatment cost difference (euros)	−12,792		−9080 (29)		−10,959 (14)		−11,026 (16)		−10,769 (16)	
SE cost difference	6086		8463 (39)		6853 (13)		6988 (15)		6954 (14)	
*z* for CCA and *t* for MI	−2.100		−1.070		−1.600		−1.580		−1.550	
*p* value	0.036		0.283		0.110		0.115		0.122	
95 % CI lower limit	−24,720		−25,667		−24,393		−24,725		−24,400	
95 % CI upper limit	−865		7508		2475		2673		2862	
*50* *% Missing data*										
*n*	237	193	132	91	237	193	237	193	237	193
Mean cost (euros)	50,560	37,767	50,160	42,794	48,711	38,335	49,180	38,527	49,110	39,454
SE mean (euros)	5359	3063	7447	5336	5875	3513	5857	3501	5913	3683
Treatment cost difference (euros)	−12,792		−7366 (42)		−10,376 (19)		−10,653 (17)		−9656 (25)	
SE cost difference	6086		9496 (56)		6852 (13)		6764 (11)		6954 (14)	
*z* for CCA and *t* for MI	−2.100		−0.780		−1.510		−1.570		−1.390	
*p* value	0.036		0.438		0.130		0.115		0.165	
95 % CI lower limit	−24,720		−25,978		−23,810		−23,912		−23,289	
95 % CI upper limit	−865		11,246		3058		2607		3977	

*M* refers to the methadone maintenance treatment group, *M* *+* *H* refers to the group that had medical co-prescription of heroin. *SE* standard error, *CI* confidence interval, *RA* reference analysis, *CCA* complete case analysis, *PMM* multiple imputation with predictive mean matching, *Log MI-PMM* multiple imputation with predictive mean matching on log-transformed costs, *Two-step-MI* two-step multiple imputation with predictive mean matching

### QALYs

Table 4 provides the QALY results for the 17, 35 and 50 % missing data. In the 17 % missing data set, all strategy deviations were roughly the same amount for the difference in QALYs and standard error. All imputation strategies, including the CCA, showed a statistically significant difference (*p* < 0.001) in QALYs between the two intervention groups.

**Table 4:** Overview clinical effect estimates of QALY model for the missing data methods

	RA		CCA (% bias)		MI-PMM (% bias)		Log MI-PMM (% bias)		Two-step MI (% bias)	
	M	M + H	M	M + H	M	M + H	M	M + H	M	M + H
*17* *% Missing data*										
*n*	237	193	201	154	237	193	237	193	237	193
Mean (QALY)	0.730	0.798	0.722	0.798	0.728	0.792	0.727	0.791	0.728	0.792
SE mean (QALY)	0.015	0.016	0.017	0.016	0.016	0.016	0.016	0.016	0.020	0.016
QALY difference	0.054		0.060 (11)		0.061 (12)		0.061 (12)		0.061 (12)	
SE QALY difference	0.018		0.020 (12)		0.020 (10)		0.020 (10)		0.020 (11)	
*z* for CCA and t for MI	2.970		2.950		3.020		3.020		3.000	
*p* value	0.003		0.003		0.003		0.003		0.003	
95 % CI lower limit	0.018		0.020		0.021		0.021		0.021	
95 % CI upper limit	0.090		0.100		0.100		0.100		0.100	
*35* *% Missing data*										
*n*	237	193	163	122	237	193	237	193	237	193
Mean (QALY)	0.730	0.790	0.715	0.790	0.718	0.790	0.717	0.791	0.718	0.790
SE mean (QALY)	0.015	0.018	0.020	0.018	0.017	0.016	0.017	0.016	0.017	0.016
QALY difference	0.054		0.068 (24)		0.069 (27)		0.071 (30)		0.069 (27)	
SE QALY difference	0.018		0.023 (27)		0.021 (17)		0.022 (18)		0.022 (20)	
*z* for CCA and t for MI	2.970		2.910		3.230		3.260		3.150	
*p* value	0.003		0.004		0.001		0.001		0.002	
95 % CI lower limit	0.018		0.022		0.027		0.028		0.026	
95 % CI upper limit	0.090		0.113		0.111		0.113		0.112	
*50* *% missing data*										
*n*	237	193	132	91	237	193	237	193	237	193
Mean (QALY)	0.730	0.782	0.717	0.782	0.705	0.785	0.708	0.784	0.706	0.784
SE mean (QALY)	0.015	0.021	0.021	0.021	0.018	0.017	0.018	0.018	0.018	0.018
QALY difference	0.054		0.047 (13)		0.077 (41)		0.074 (36)		0.075 (38)	
SE QALY difference	0.018		0.026 (43)		0.024 (29)		0.024 (30)		0.024 (31)	
*z* for CCA and t for MI	2.970		1.820		3.260		3.110		3.140	
*p* value	0.003		0.069		0.001		0.002		0.002	
95 % CI lower limit	0.018		−0.004		0.031		0.027		0.028	
95 % CI upper limit	0.090		0.098		0.123		0.120		0.122	

*M* refers to the methadone maintenance treatment group, *M* *+* *H* refers to the group that had medical co-prescription of heroin. *SE* standard error, *QALY* quality of life years gained, *CI* confidence interval, *RA* reference analysis, *CCA* complete case analysis, *PMM* multiple imputation with predictive mean matching, *Log MI-PMM* multiple imputation with predictive mean matching on log-transformed costs, *Two-step-MI* two-step multiple imputation with predictive mean matching

In the data set with 35 % missing data, the QALY coefficient in the CCA deviated the least and the most deviation occurred in the log MI-PMM, but the reference coefficient was still contained in all confidence intervals. The standard error of the CCA deviated the most from the standard error in the RA while the MI-PMM deviated the least. All strategies still showed that co-prescribed heroin was associated with higher QALY scores compared to methadone maintenance.

In the 50 % missing data set, the QALY coefficient deviated the most in the MI-PMM and the least in the CCA but the regression coefficient from the RA was still within all 95 % confidence intervals. The standard error for the CCA deviated the most from the reference analysis, but the deviation in all MI strategies was similar. The CCA was the only strategy where the difference in QALYs was no longer statistically significant.

### Cost-utility analysis

**Fig. 1:**
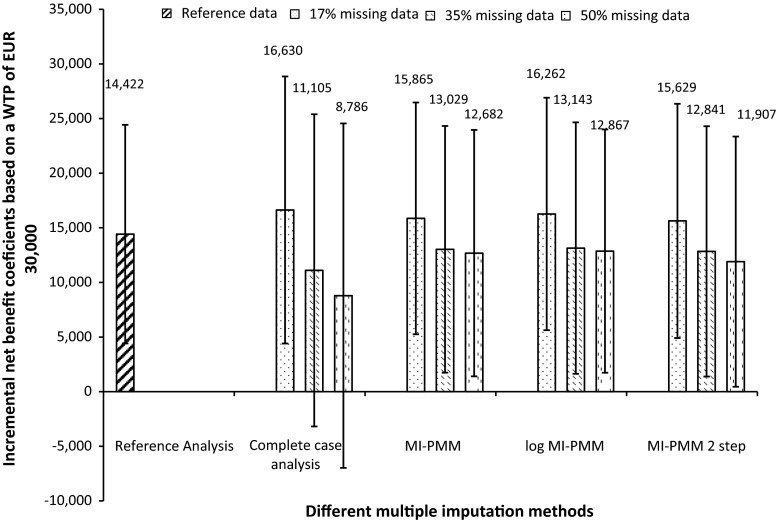
Incremental net benefit (in euros) coefficients for a threshold value of €30,000 based on the amount of missing data and imputation method. *MI-PMM* multiple imputation with predictive mean matching, *log-MI-PMM* multiple imputation with predictive mean matching on log-transformed costs, *MI-PMM 2 step* two-step multiple imputation with predictive mean matching

Figure 1 and Table 5 show the ICERs, INB, its variance, and the probability that co-prescribed heroin compared to methadone maintenance is cost-effective at a threshold value of €30,000/QALY for the 17, 35 and 50 % missing data sets. The CCA showed the largest deviation from the RA for the INB and its standard error, and the ICER in the 17 % missing data scenario. The INBs in the two-step MI strategy deviated the least from the INB in the reference analysis. The standard error deviated similarly for all imputation strategies. The reference value of INB was contained in the confidence intervals of all imputation strategies. The probability of co-prescribed heroin compared to methadone maintenance being cost-effective was 99 % for a willingness-to-pay threshold value of €30,000 for a one-unit gain in QALY score regardless of the imputation strategy.

**Table 5:** Cost effectiveness analysis estimates for the missing data methods

	RA	CCA (% bias)	MI-PMM (% bias)	Log MI-PMM (% bias)	Two-step MI (% bias)
*17* *% Missing data*					
Correlation utility and costs	0.0507	0.0591	0.0517	0.0509	0.0487
Covariance	5.6	8.6	6.7	6.6	6.4
Mean (INB)	14,422	16,026 (11)	15,257 (6)	15,654 (9)	15,023 (4)
SE INB	6083	7270 (20)	6438 (6)	6457 (6)	6504 (7)
95 % CI lower limit	4417	4069	4669	5034	4324
95 % CI upper limit	24,427	27,983	25,846	26,274	25,721
Prob C-E	0.99	0.99 (0)	0.99 (0)	0.99 (0)	0.99 (0)
ICER	−235,472	−235,448 (0)	−220,988 (6)	−227,410 (3)	−217,656 (8)
*35* *% Missing data*					
Correlation utility and costs	0.0507	0.0251	0.0300	0.0292	0.028
Covariance	5.6	4.9	4.4	4.4	4.4
Mean (INB)	14,422	11,105 (23)	13,029 (10)	13,143 (9)	12,841 (11)
SE (INB)	6083	8685 (43)	6864 (13)	7000 (15)	6966 (15)
95 % CI lower limit	4417	−3181	1738	1629	1383
95 % CI upper limit	24,427	25,390	24,319	24,656	24,299
Prob C-E	0.99	0.90 (9)	0.97 (2)	0.97 (2)	0.97 (2)
ICER	−235,472	−134,488 (43)	−158,857 (33)	−156,289 (34)	−155,935 (34)
*50* *% Missing data*					
Correlation utility and costs	0.0507	0.0223	0.0433	0.0436	0.0406
Covariance	5.6	5.5	7.0	7.0	6.7
Mean (INB)	14,422	8786 (39)	12,682 (12)	12,867 (11)	11,907 (17)
SE (INB)	6083	9584 (58)	6858 (13)	6770 (11)	6962 (14)
95 % CI lower limit	4417	−6978	1401	1731	456
95 % CI upper limit	24,427	24,551	23,962	24,003	23,358
Prob C-E	0.99	0.82 (17)	0.97 (2)	0.97 (2)	0.96 (3)
ICER	−235,472	−155,561 (34)	−134,979 (43)	−144,317 (39)	−128,670 (45)

*SE* standard error, *INB* incremental net benefit (euros), *CI* confidence interval, *Prob C-E* probability of cost-effectiveness, *ICER* incremental cost effectiveness ratio

In the 35 % missing data scenario, the CCA deviated the most from the RA for the ICER, INB and its standard error, and the probability that the intervention was cost effective. The MI-PMM deviated least from the RA for the INB standard error compared to the other imputation strategies. The probability of co-prescribed heroin being cost-effective compared with methadone maintenance was 97 % for a willingness-to-pay threshold value of €30,000 for a one-unit gain in QALY score for all multiple imputation strategies versus 99 % for the RA (CCA was 90 %).

In the scenario with 50 % missing, the INB was no longer statistically significant for the CCA. The log MI-PMM showed the least deviation from the RA in the INB coefficient and its standard error, and the probability that the intervention was cost effective. The probability of co-prescribed methadone being cost-effective compared with methadone maintenance at €30,000/QALY was 97 %.

**Fig. 2:**
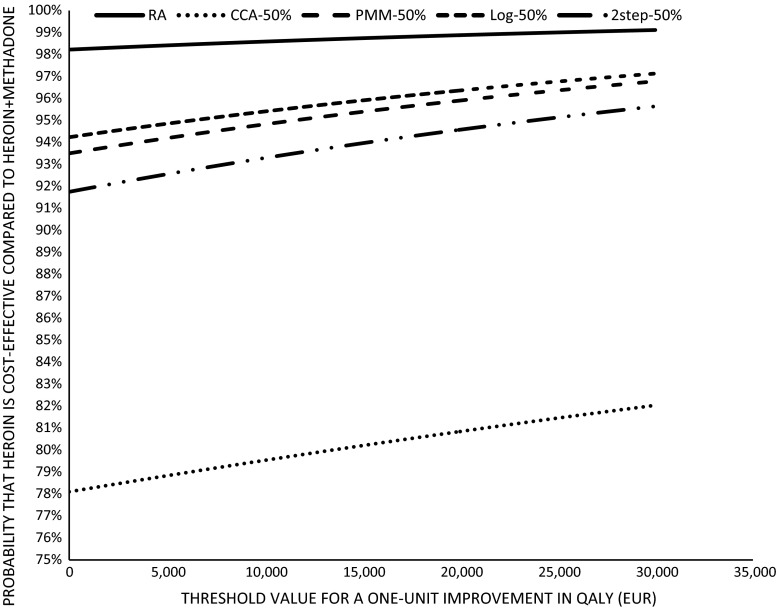
Cost-effectiveness acceptability curves for threshold value of €30,000 based on the 50 % missing data scenario. *PMM* Multiple imputation with predictive mean matching, *Log* multiple imputation with predictive mean matching on log-transformed costs, *2step* two-step multiple imputation with predictive mean matching

The reference INB was within the 95 % confidence intervals for all imputation strategies (see Fig. 1). For the CCA, INB was no longer statistically significant with 35 and 50 % missing data. INB decreased with higher rates of missing data and the uncertainty was larger, as evidenced by the larger standard errors and wider confidence intervals in all strategies. The log MI-PMM showed the least uncertainty around INB in all missing data scenarios. Figure 2 presents the CEAC curves for the different strategies with 50 % missing data. This figure shows that there are pronounced differences between the strategies in this scenario. It shows that the probability that co-prescribed heroin is cost-effective when the threshold value is zero is 98 % for the reference analysis, 94 % for the log MI-PMM and MI-PMM, 92 % for the two-step-MI and 78 % for the CCA. This increases to 99, 97, 97,96 and 82 % for the RA, MI-PMM, log MI-PMM, two-step MI and CCA, respectively, at a threshold value of €30,000/QALY.

### Sensitivity analysis

The imputation procedure was applied to the total costs directly for the MI-PMM and the log MI-PMM. The results showed that the precision decreased, resulting in wider standard errors and increased percentage of bias in the cost difference from the reference analysis when applying multiple imputation to the total costs compared with imputation of sub-cost variables (data not shown).

## Discussion

### Main findings

In this study, we evaluated the performance of different multiple imputation strategies and CCA for scenarios with varying rates of missing data in costs and effects in a pragmatic economic evaluation. We found that for all rates of missing data, multiple imputation strategies performed better than CCA. The results of the CCA, MI-PMM and the two-step MI were all influenced by the amount of missing data. With a larger amount of missing data, the log MI-PMM deviated the least from the RA for the cost difference, cost standard error, INB and its standard error, and the probability that the co-prescribed heroin treatment was cost effective in comparison with methadone maintenance at a willingness to pay of €30,000 per QALY. Therefore, the log MI-PMM is considered most appropriate for imputing missing cost and effect data. However, when considering QALYs the MI-PMM performed best since it deviated the least from the RA with increasing amounts of missing data. Overall, the log MI-PMM was least affected by the amount of missing data.

Our results imply that addressing only the right-skewness of the data by using a log transformation in combination with PMM is enough and that strategies to deal with zero inflation such as our two-step PMM are not needed. The results are also consistent with the advice in the literature that recommends implementing a log transformation when imputing skewed data [4, 5, 8].

Beforehand, we expected that the two-step MI strategy would have performed better because it controls for the large amount of zeroes and the skewness in the data. However, in practice there were no relevant differences with the other multiple imputation strategies and the two-step MI was more difficult to apply than the log MI-PMM. Not all software packages have incorporated a comprehensive way to apply the two-step MI strategy, whereas the log MI-PMM is easily applied and available in software packages like SPSS, Stata, SAS and R.

### Comparison with existing literature

Our study adds to the findings from other studies that multiple imputation is better than CCA for dealing with missing data in economic evaluations [2, 3, 8, 28, 29]. However, in contrast to Briggs et al. [3], Oostenbrink et al. [2] and Burton et al. [28], we had information on the observed values of the missing data, because we created the missing data ourselves using the MAR assumption. This allowed us to estimate the deviation of the different imputation strategies from the original complete data set.

Yu et al. [29] showed in a simulation study that predictive mean matching in R and STATA performed reasonably well and maintained the underlying distribution of the resource use data [29]. However, they did not evaluate the effect of the different imputation strategies on the cost-effectiveness estimates.

Faria et al. [30] created a structured approach and practical guidance on how to handle missing data on costs and health outcomes while comparing inverse probability weighting, multiple imputation and likelihood-based methods. They concluded that multiple imputation was flexible to use and allowed for more flexible sensitivity analyses. They did not look at the different types of multiple imputation strategies that we have in economic evaluations.

### Strengths and limitations

Our study adds to previous studies by focussing on estimation of both incomplete costs, QALYs and cost-effectiveness and by comparing different MI strategies using the MICE (PMM) in STATA. Additionally, we use a correlation after multiple imputation between costs and utilities using Fisher’s Z transformation to calculate the cost-effectiveness [5, 22]. We used the fully conditional specification with PMM which gave us more flexibility around assumptions of normality [31].

Other strengths of this study were its systematic and applied approach using real data to examine the performance of different multiple imputation strategies in situations with varying amounts of missing data. To our knowledge, this is one of the first studies to compare the two-step MI strategy with other multiple imputation strategies for cost-effectiveness evaluations.

As we used only one data set we were limited in our evaluation parameters for direct comparisons to the true coefficients instead of averages over simulations. We did perform a small simulation pilot study repeating the imputation procedures to verify the stability of the methods. This was done by repeatedly drawing samples of 100 cases from each of our incomplete data sets and applying our method to these small samples. We simulated 1000 times and used 15 imputations and 20 iterations. For each method and incomplete data condition the average over the 1000 simulations was taken and compared to the complete reference data results. This simulation confirmed the relative differences between the performances of the methods presented in this study. This might reduce generalizability to other scenarios and contexts. Future research should perform a larger simulation study and vary the proportion of zeroes to see how that affects the performance of the missing data methods. It is possible that with a greater amount of zeroes the two-part model becomes more beneficial over the other methods. We assumed the same missing mechanism in both treatment arms, and in future simulations this probably should be changed using simulated data.

### Implications for further research

Prospective economic evaluations alongside trials play an important role in providing decision makers with cost-effectiveness information to inform reimbursement decisions. It is important that economic evaluations provide robust and unbiased information. The consequences of using different imputation strategies can affect policy decisions. In this study, we considered co-prescribed heroin treatment to be cost-effective in comparison with methadone maintenance in all strategies evaluated, although the uncertainty increased. The decision may change depending on the imputation procedure chosen in situations with smaller differences between groups.

In conclusion, we recommend the use of the log MI-PMM because of its ease of use and its reliable results with increasing amounts of missing data. Log MI-PMM also appears to perform well for zero-inflated data, providing a constant is used in place of the zero in the data.
